# Seismic performance and bearing capacity calculation of cross shaped concrete columns with built-in T-shaped steel and steel tubes

**DOI:** 10.1371/journal.pone.0290426

**Published:** 2023-11-17

**Authors:** Lidong Zhao, Yong Yu, Congrong Tang, Licai Zhong, Qirong Qiu

**Affiliations:** 1 College of Computer and Artificial Intelligence, LanZhou Institute of Technology, Lanzhou, Gansu Province, China; 2 School of Environment and Civil Engineering, Dongguan University of Technology, Dongguan, Guangdong Province, China; 3 Nanjing Sutong Road Bridge Engineering Co., Ltd., Nanjing, Jiangsu Province, China; 4 Lanzhou Resources & Environment Voc—Tech University, Lanzhou, Gansu Province, China; 5 Shanghai Construction Engineering Fifth Construction Group Co., Ltd., Shanghai, China; University of Vigo, SPAIN

## Abstract

Incorporating T-shaped steel and square steel tubes into a cross shaped concrete column can significantly improve the seismic performance of the cross shaped column. However, the experimental samples are limited, so ABAQUS finite element (FE) analysis method was adopted in this paper to study the seismic performance of this cross shaped column, calculate and verify three specimens in the existing reference. Based on the reliable model, parameter analysis was carried out (25 specimens in total). The results show that the established model has a high degree of coincidence in the hysteretic curve, skeleton curve and failure mode, and the error of ultimate bearing capacity and ductility is within 10%. The configuration of T-shaped steel and square steel tubes inside the cross column can meet the ductility requirements specified in the standard under high axial compression ratio. The ultimate bearing capacity of the cross shaped column increases with the increase of the thickness of the square steel tube, but the ductility deteriorates. The increase in steel tube size increases the strength of the concrete in the core area, and the seismic performance of the cross shaped column was improved. Increasing the thickness of the T-shaped steel flange can better improve the seismic performance of the cross shaped column compared to increasing the thickness of the T-shaped steel web plate. Increasing the height of the specimen will significantly reduce its seismic performance. When the shear span ratio is not greater than 4.1, the ductility can meet the standard requirements. The error of the formula for calculating the compression-bending bearing capacity proposed based on existing calculation methods is less than 5%.

## Introduction

The special-shaped column structural system is a novel structural system that can be formed into various shapes, such as cross, T, L, or other combination shapes, based on specific building locations. These specially shaped columns can eliminate protruding column edges, simplify cross-sectional dimensions, increase usable floor area, and provide flexible layouts compared to traditional reinforced concrete columns [1–5]. However, in practice, reinforced concrete special-shaped columns often exhibit low stiffness, bearing capacity, and seismic performance due to their reduced cross-sectional area. Therefore, in recent years, steel-concrete composite special-shaped columns (SRC) have been proposed, effectively combining steel and concrete to overcome these drawbacks. Compared with ordinary cross shaped concrete columns, steel cross shaped concrete columns have the following advantages: 1) The steel web plate constrains the concrete core, improving the compressive strength and ductility of the concrete; 2) The cross shaped section has a large perimeter ratio and cross-sectional moment of inertia, which is beneficial for improving its ability to resist lateral displacement; 3) The steel web plate has strong resistance to local damage and can effectively improve the problem of reduced bearing capacity of concrete after earthquakes. Therefore, the steel cross shaped concrete column is expected to exhibit better seismic performance than traditional concrete columns.

**Fig 1:**
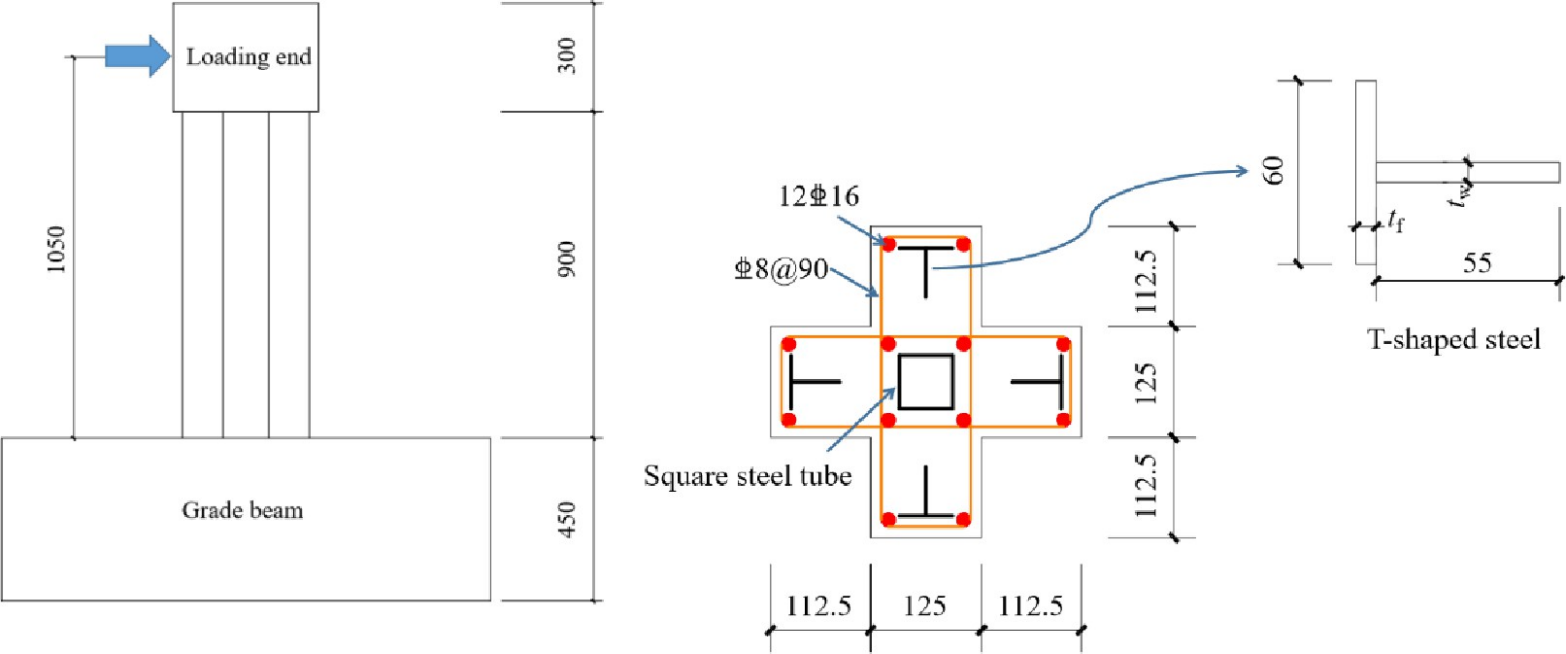
Structural diagram of specimen.

Significant progress has been made in studying the mechanical properties of SRC special-shaped columns [6–12]. The presence of shaped steel improves the ductility and load-bearing capacity of SRC special-shaped columns. However, most existing literature focuses on solid-web or open-web section steel, with limited research on combinations of section steel and steel tubes. Li [13] tested the seismic performance of 5 cross-shaped columns with embedded T-shaped steel and square steel tubes (Fig 1). Results showed that using T-shaped steel and square steel tubes inside cross-shaped columns can effectively improve section stiffness and seismic performance compared to solid web SRC cross-shaped columns. Therefore, T-shaped steel plus square steel tubes show promise as an optimized steel configuration for cross-shaped special-shaped columns. However, existing research on this steel configuration has only considered different axial compression ratios as variables. Therefore, in order to further promote the research of this composite column, it is necessary to conduct more parameter analysis.

In order to further study the cross shaped concrete column with built-in T-shaped steel and steel tubes, ABAQUS software is adopted to simulate and verify the experimental results of Li [13]. Based on a reliable model, in-depth analysis was conducted on 28 samples (including 3 samples and 25 numerical simulation analysis samples). The influence of different variables on the seismic performance of cross shaped concrete columns with built-in T-shaped steel and steel tubes is discussed. According to the existing calculation methods, the calculation method of the compression-bending bearing capacity of SRC cross shaped columns is proposed.

## Experimental program

Li [13] designed and made three cross shaped concrete columns with built-in T-shaped steel and steel tubes with axial compression ratio as the variable parameter, and the size and steel design of the cross shaped column are shown in Fig 1. The test section length of all SRC cross shaped columns was 900mm, the height from the center of the electro-hydraulic servo actuator to the surface of the ground beam was designed to be 1050mm. Detailed design parameters of test specimen and extended analysis specimen are shown in Table 1. The yield strength *f*_y_ = 405MPa of T-shaped steel and steel tube adopted in the test, and the ultimate strength *f*_u_ = 538MPa. Chinese standard C40 grade concrete was adopted, and the measured compressive strength of the concrete cube was 41.1MPa.

**Table 1:** Specimen design parameters and results.

Specimen No.	*n*	*t*/mm	*d*/mm	*t*_f_/mm	*t*_w_/mm	*H*/mm	*P*_p_/kN	*μ*	*P*_c_/kN	Remarks
SRC-2	0.19	6	60	6	6	1050	195.7	3.56	185.9	Reference [13]
SRC-3	0.24	6	60	6	6	1050	205.2	3.28	215.5	
SRC-4	0.34	6	60	6	6	1050	207.1	4.58	196.7	
E-1	0.34	6	60	6	6	1050	203.2	4.86	205.2	Change of axial pressure ratio
E-2	0.4	6	60	6	6	1050	200.8	5.02	208.8	
E-3	0.5	6	60	6	6	1050	195.2	3.87	195.2	
E-4	0.6	6	60	6	6	1050	186.5	3.97	184.6	
E-5	0.7	6	60	6	6	1050	175.0	3.80	168	
E-6	0.34	4	60	6	6	1050	199.7	4.72	199.7	Steel tube thickness change
E-7	0.34	5	60	6	6	1050	201.8	4.78	211.9	
E-8	0.34	7	60	6	6	1050	205.3	4.92	213.5	
E-9	0.34	8	60	6	6	1050	206.8	5.01	208.9	
E-10	0.34	6	60	4	6	1050	190.9	4.49	196.6	Thickness change of T-shaped steel flange
E-11	0.34	6	60	5	6	1050	198.0	4.70	194	
E-12	0.34	6	60	7	6	1050	211.3	4.97	217.6	
E-13	0.34	6	60	8	6	1050	218.7	5.16	207.8	
E-14	0.34	6	60	6	4	1050	194.6	4.64	186.8	Thickness change of T-shaped steel web
E-15	0.34	6	60	6	5	1050	199.6	4.75	195.6	
E-16	0.34	6	60	6	7	1050	208.8	4.93	215.1	
E-17	0.34	6	60	6	8	1050	213.5	5.03	222	
E-18	0.34	6	40	6	6	1050	185.0	4.42	181.3	Change of side length of steel tube
E-19	0.34	6	50	6	6	1050	193.6	4.56	183.9	
E-20	0.34	6	70	6	6	1050	212.0	5.32	205.6	
E-21	0.34	6	80	6	6	1050	217.0	5.91	212.7	
E-22	0.34	6	60	6	6	1250	188.3	3.94	183.4	Changes in height
E-23	0.34	6	60	6	6	1450	160.5	3.16	155.7	
E-24	0.34	6	60	6	6	1650	136.4	2.63	135.2	
E-25	0.34	6	60	6	6	1850	118.3	2.23	111.1	

During testing, the designed axial load was first applied to the top of the column using a hydraulic jack and kept constant throughout loading. After reaching the predetermined axial load value, horizontal load was applied through an MTS electro-hydraulic servo actuator. A load-displacement joint control loading scheme was used, with load control before yield and displacement control after yield. Prior to yield, loading was applied in 60kN increments until the specimen reached yield. After yield, loading and unloading at each stage was repeated three times under displacement control.

## FE analysis

### Materials

#### Steel

**Fig 2:**
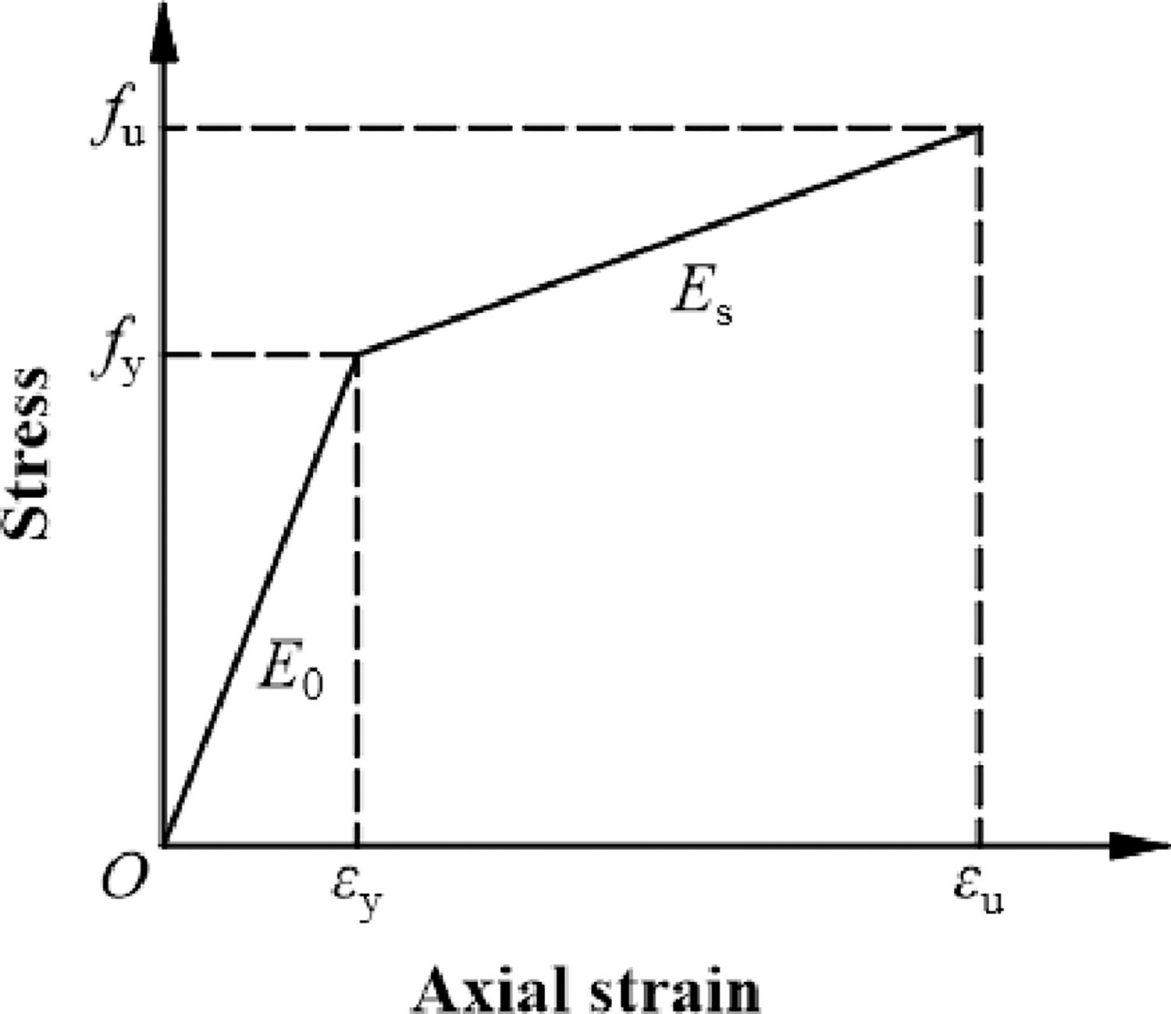
Elastic-plastic model.

The bilinear model of strain hardening was adopted in ABAQUS/CAE, without considering large deformation of the material. **Eq 1** provides the calculation formula for the constitutive relationship of steel. The constitutive relationship curve is shown in Fig 2. The required values for ABAQUS were obtained through experimental measurements [14, 15].


$$\sigma =\{\begin{array}{ll} E_{0}\varepsilon & \left(0\leq \varepsilon \leq \varepsilon_{y}\right)\\ f_{y}+E_{s}(\varepsilon -\varepsilon_{y}) & \left(\varepsilon_{y}\leq \varepsilon \right) \end{array}$$ (1)

#### Concrete

**Fig 3:**
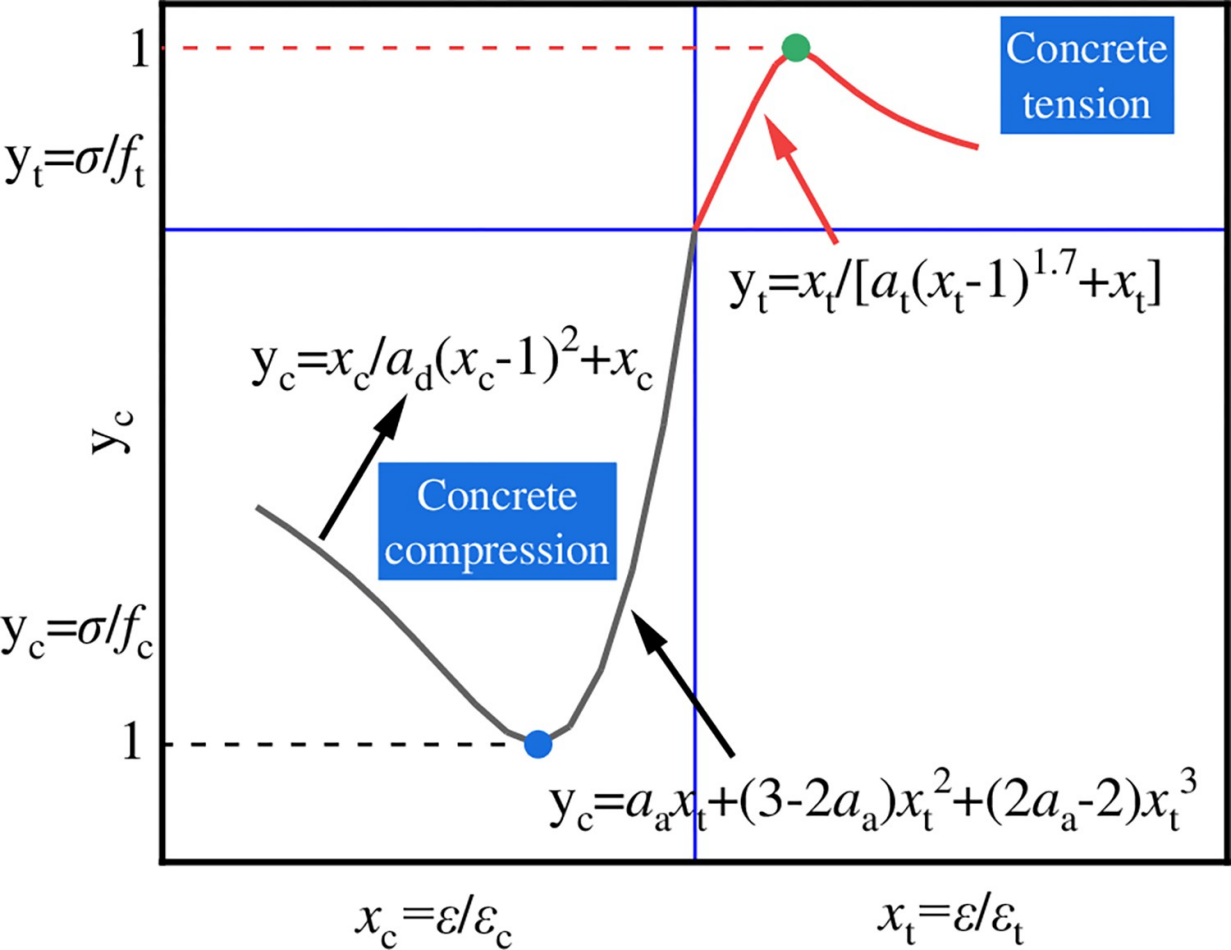
CDP model.

In this paper, the concrete plastic damage model (CDP) was adopted. Ordinary commercial concrete was adopted in the experiment, so the stress-strain relationship curve of concrete specified in the standard can be adopted [16]. The schematic diagram and calculation formula of the CDP model are shown in Fig 3. The strength grade of concrete in this paper was C40, and the reference standard is *α*_a_ = 2.03, *α*_d_ = 1.36 and *α*_t_ = 1.25.

### Element selection

The concrete, T-shaped steel, square steel tubes and reinforcement cage were combined into a FE model. The size of each component was consistent with the experiment, and after trial calculation, the model was divided into grids with a size of 25mm. T-shaped steel, square steel tubes and reinforcement cage were embedded in concrete, and the bond slip between them was ignored. The C3D8R element was adopted for concrete, the S4R element was adopted for T-shaped steel and square steel tubes, and the T3D2 element was adopted for steel reinforcement skeleton.

### Numerical model

**Fig 4:**
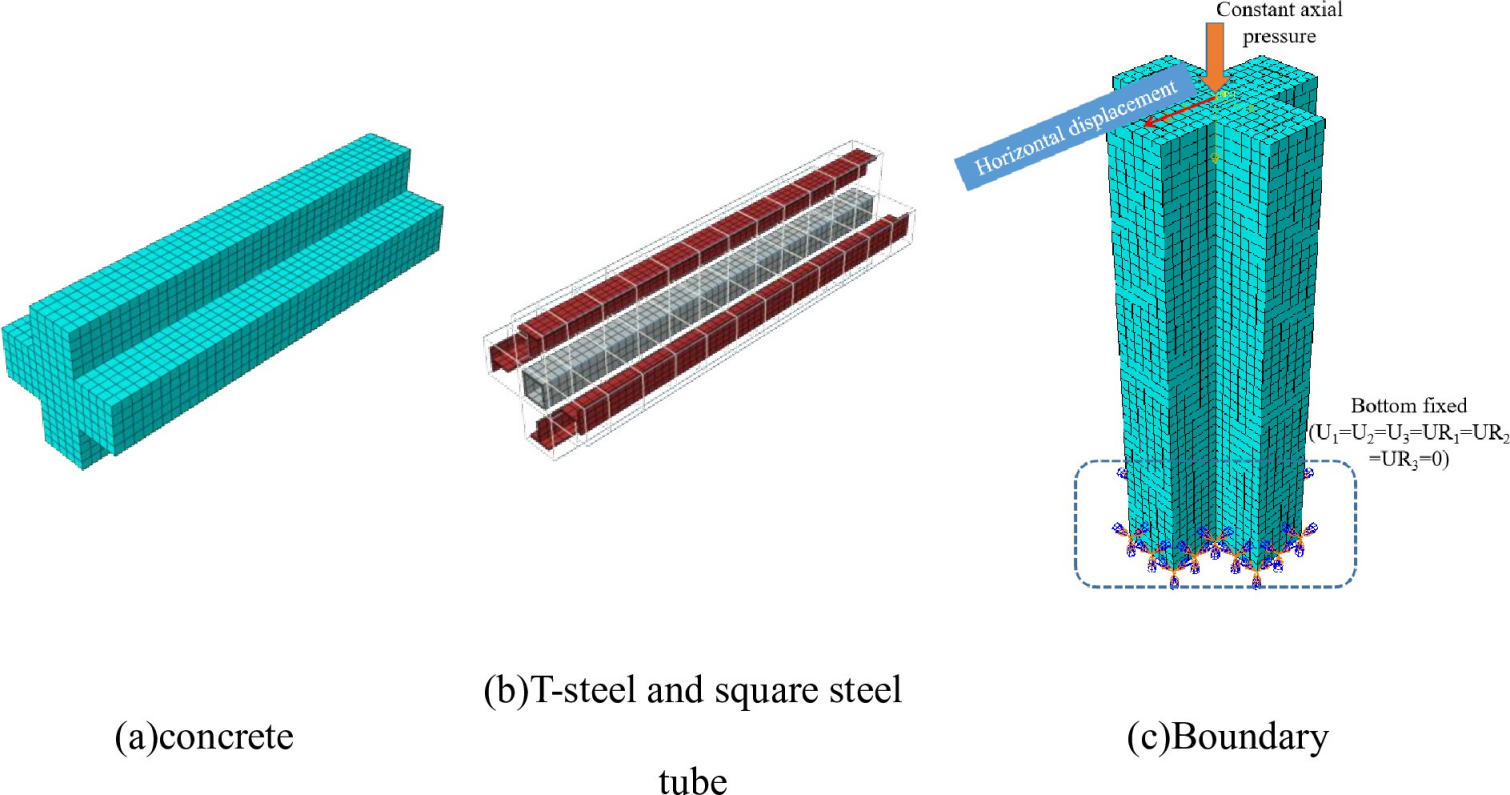
Model establishment. (a)concrete, (b)T-steel and square steel tube, (c)Boundary.

When modeling and analyzing low cycle repeated loading experiments, the load beam and ground beam can be ignored. The top of the test column was equipped with a coupling reference point that can simultaneously constrain the torsion angle and horizontal displacement. Therefore, the length of the model was calculated as 1050mm, as shown in Fig 1. Horizontal displacement and constant axial pressure were applied at the top to constrain the displacement and rotation angles in the three directions at the bottom, in order to simulate the original experimental situation. The model establishment was completed as shown in Fig 4.

### Model validation

**Fig 5:**
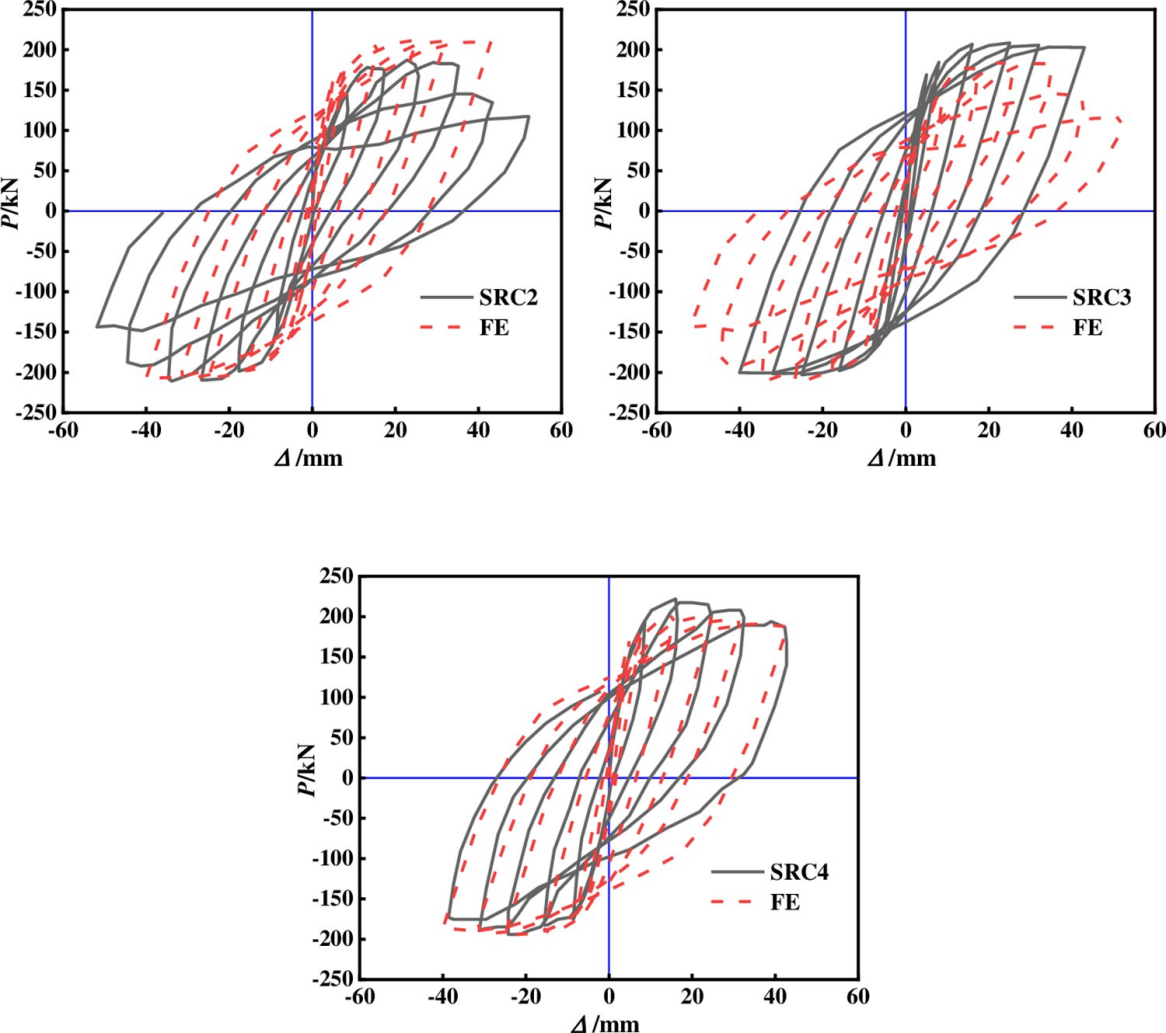
Comparison of experimental and simulated hysteresis curves.

**Fig 6:**
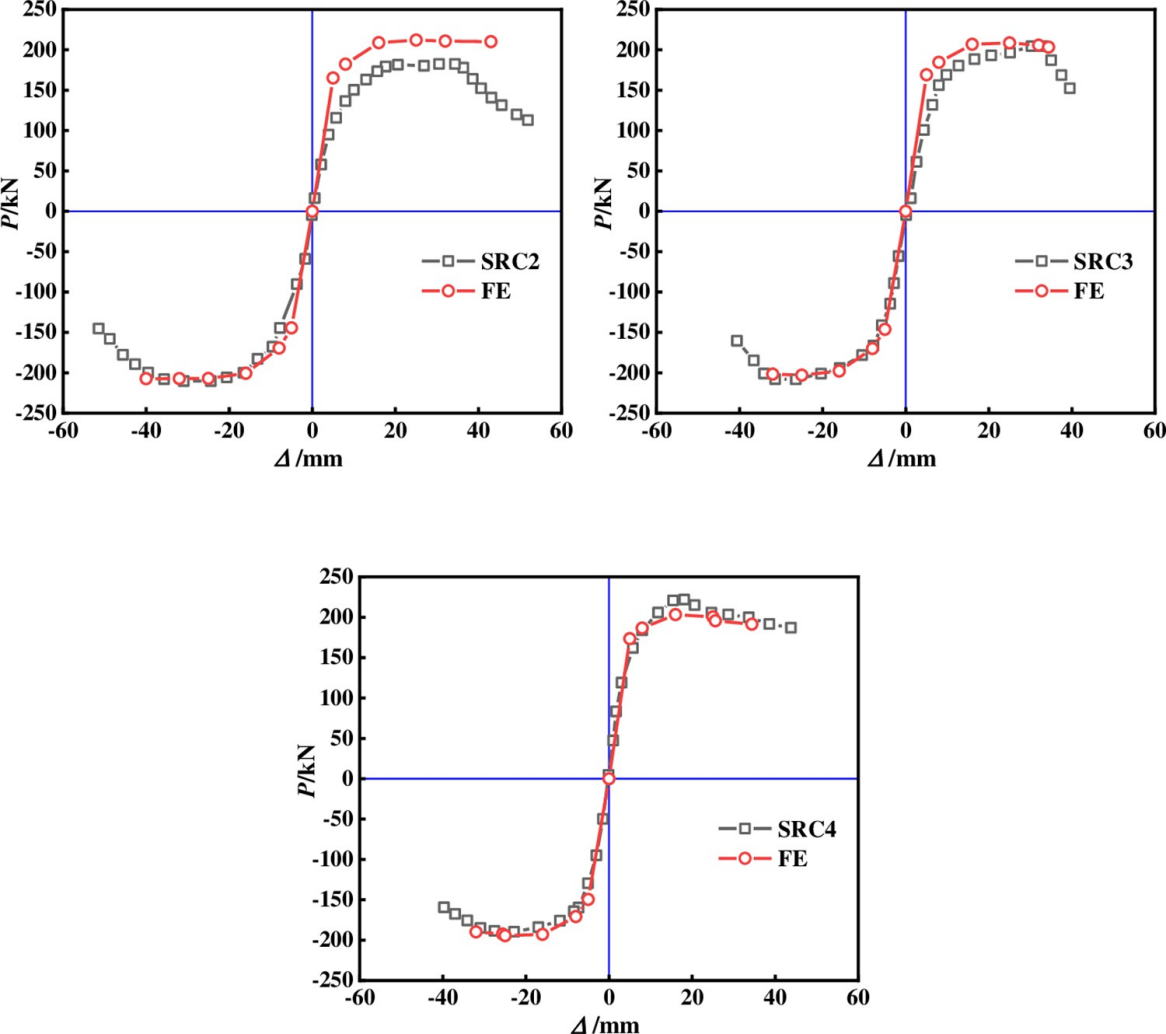
Comparison of experimental and simulated skeleton curves.

The above modeling method and material properties were adopted to simulate three cross shaped concrete columns with built-in T-shaped steel and steel tubes in Reference [13]. Figs 5 and 6 compare the hysteresis and skeleton curves of all specimens. The hysteresis curve calculated by the FE method has a pinch consistent with the experiment, and the loading and unloading stiffness of the hysteresis loop is highly consistent with the experiment, with the skeleton curve basically overlapping. There is a certain error between the bearing capacity and the initial stiffness mainly because the bond slip is not considered, and there is an inevitable error in the test, so the initial stiffness is large.

**Fig 7:**
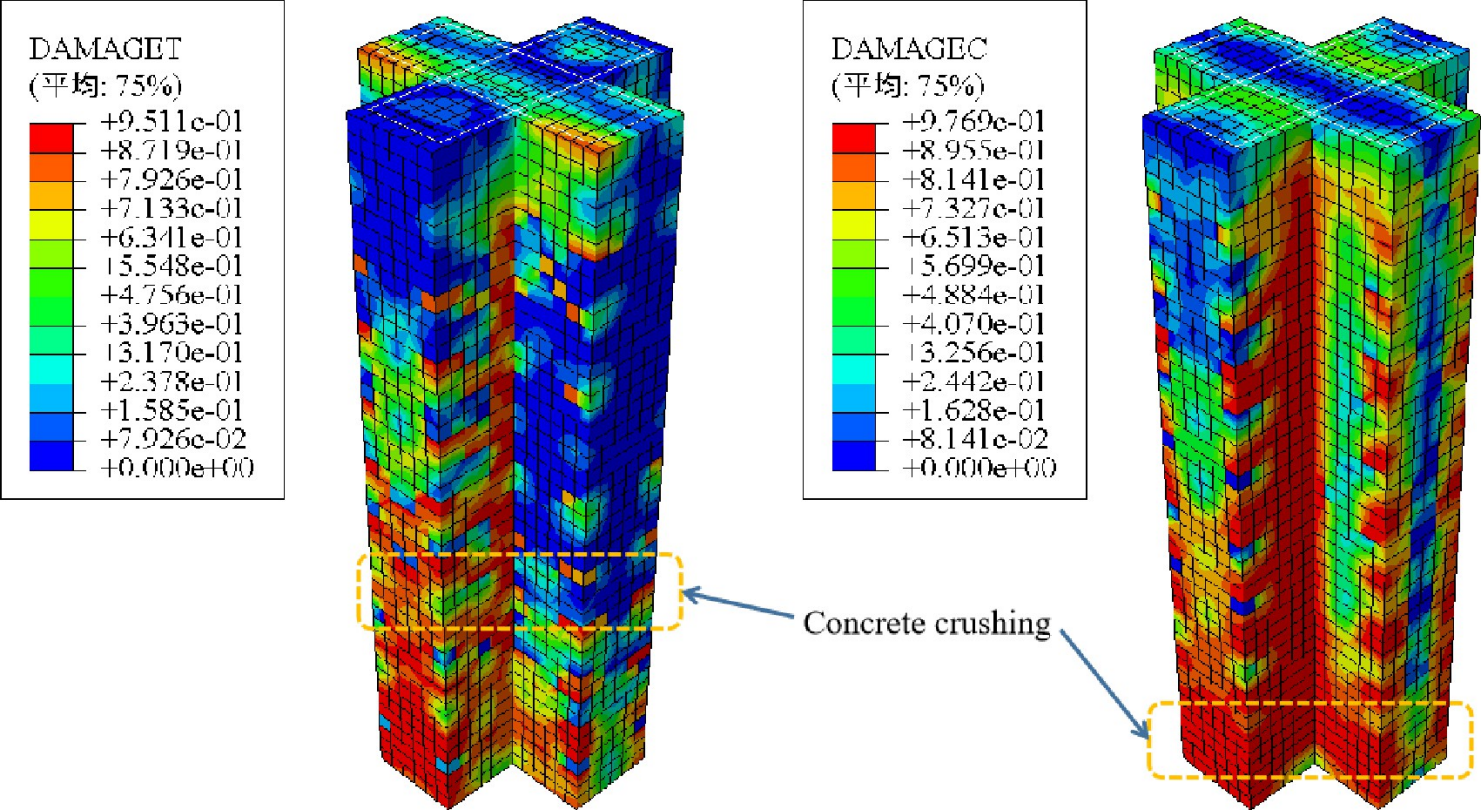
Failure modes.

Fig 7 compares the tensile damage of concrete calculated by the model with the failure mode of the specimen in the experiment. The tensile damage nephogram of concrete obtained from the FE results was very similar to the crack diagram of the test results. The concrete has interval cracks. According to the observation, the crack spacing is close to the stirrup spacing. The ultimate shear bearing capacity of SRC-2, SRC-3 and SRC-4 measured in the test are 195.7kN, 205.2kN and 207.1kN respectively, and the ductility coefficients are 3.56, 3.28 and 4.58 respectively. The FE results show that the ultimate shear bearing capacity is 210.2kN, 205.8kN and 203.2kN respectively, and the ductility coefficients are 3.68, 3.49 and 4.86 respectively, and the average error is within 10%, indicates that the model established in this article has been reliably validated.

### Stress analysis

**Fig 8:**
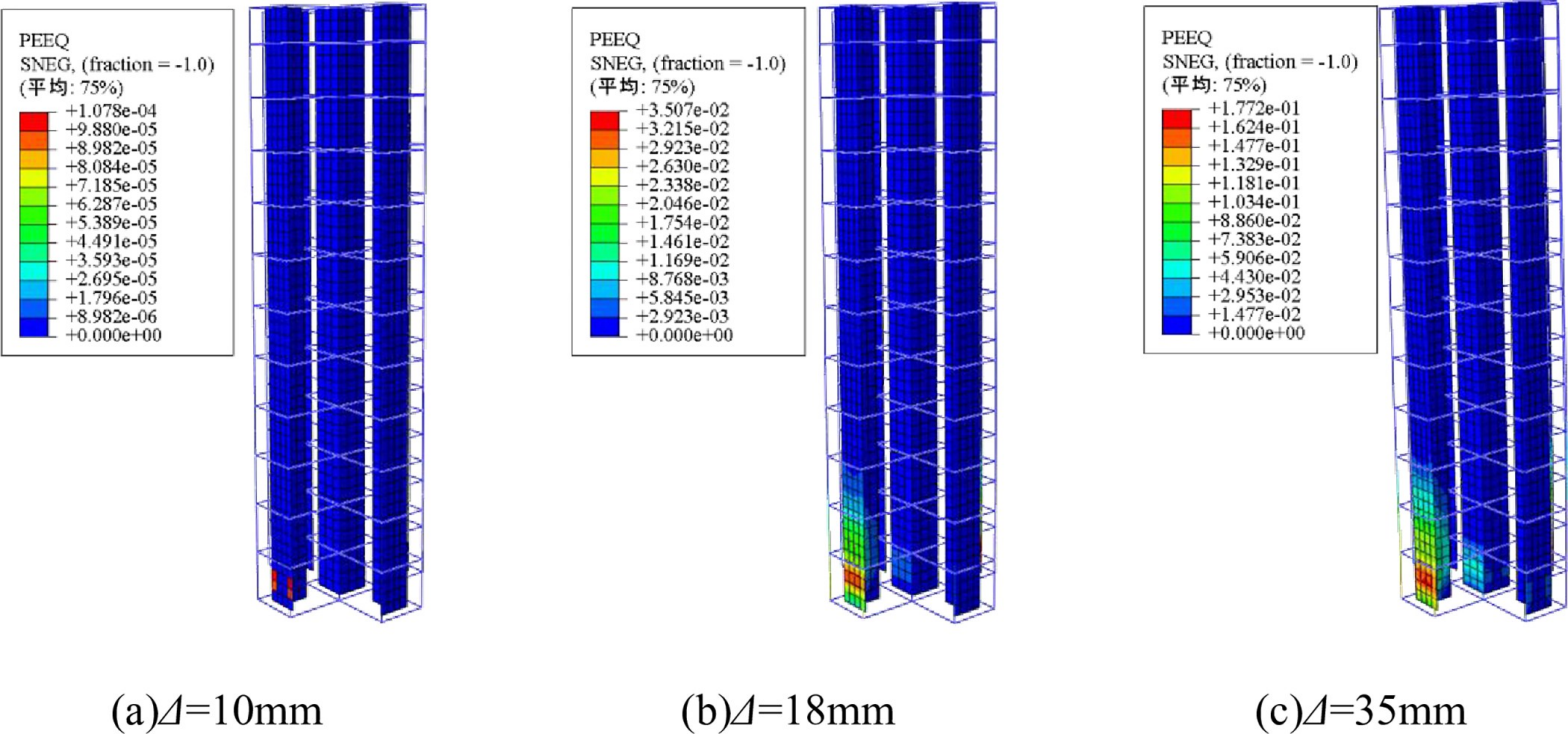
Stress development. (a)*Δ* = 10mm, (b)*Δ* = 18mm, (c)*Δ* = 35mm.

Fig 8 shows the plastic strain (PEEQ) of steel under different loading displacements. Steel yield is indicated when plastic strain occurs. As seen in Fig 8a, the steel does not yield until the elastic stage of the specimen. At approximately 10 mm displacement, the T-shaped steel flange yields first in both the compressive and tensile zones. As the limit load is reached, the longitudinal reinforcement and T-shaped steel web yield. With further increase in horizontal displacement, the steel tubes in the core area begin to yield. These results indicate that increasing the T-shaped steel flange is likely the most effective approach for improving seismic performance.

## Parameter analysis

According to the previous model modeling analysis, the FE model of the cross shaped concrete column with built-in T-shaped steel and steel tubes has been verified in terms of failure morphology and curves, and the T-shaped steel and square steel tubes inside the specimen participate in the stress to different degrees. The model of SRC-4 specimen was selected as the reference model, and 25 models were designed and calculated with the axial compression ratio *n*, the side length of the square steel tube *d*, the thickness of the square steel tube *t*, the thickness of the T-shaped steel flange *t*_f_, the thickness of the T-shaped steel web *t*_w_ and height of the specimen *H* as the variable parameters. Specific parameters and characteristic points of calculation results are shown in Table 1.

### Hysteresis curve and skeleton curve

**Fig 9:**
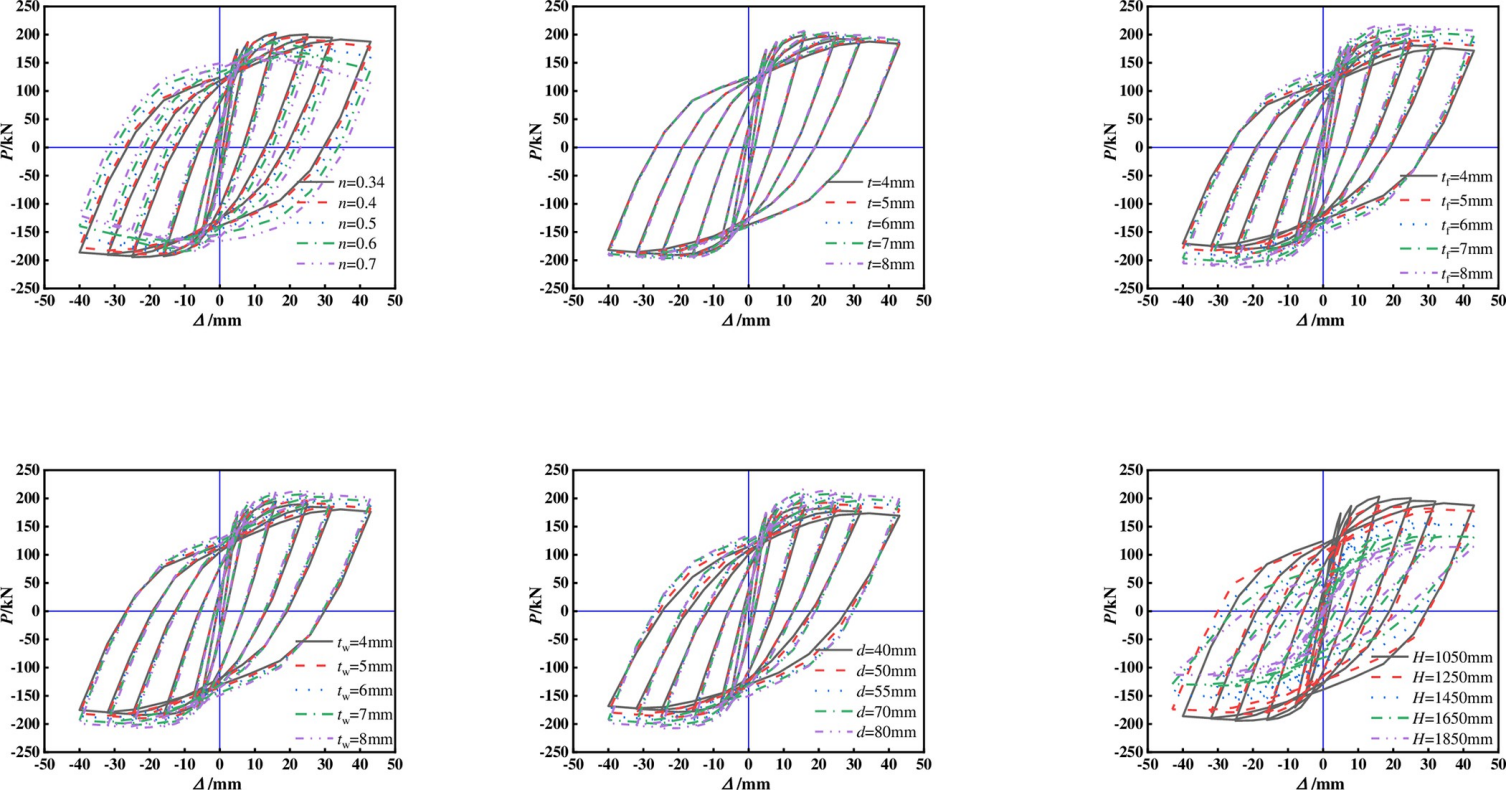
Hysteresis curve. (a)Comparison of different axial compression ratios, (b)Comparison of different square steel tube thickness, (c)Comparison of different flange thickness of T-shaped steel, (d)Comparison of different web thickness of T-shaped steel, (d)Comparison of different web thickness of T-shaped steel, (f)Comparison of different height of the specimen.

**Fig 10:**
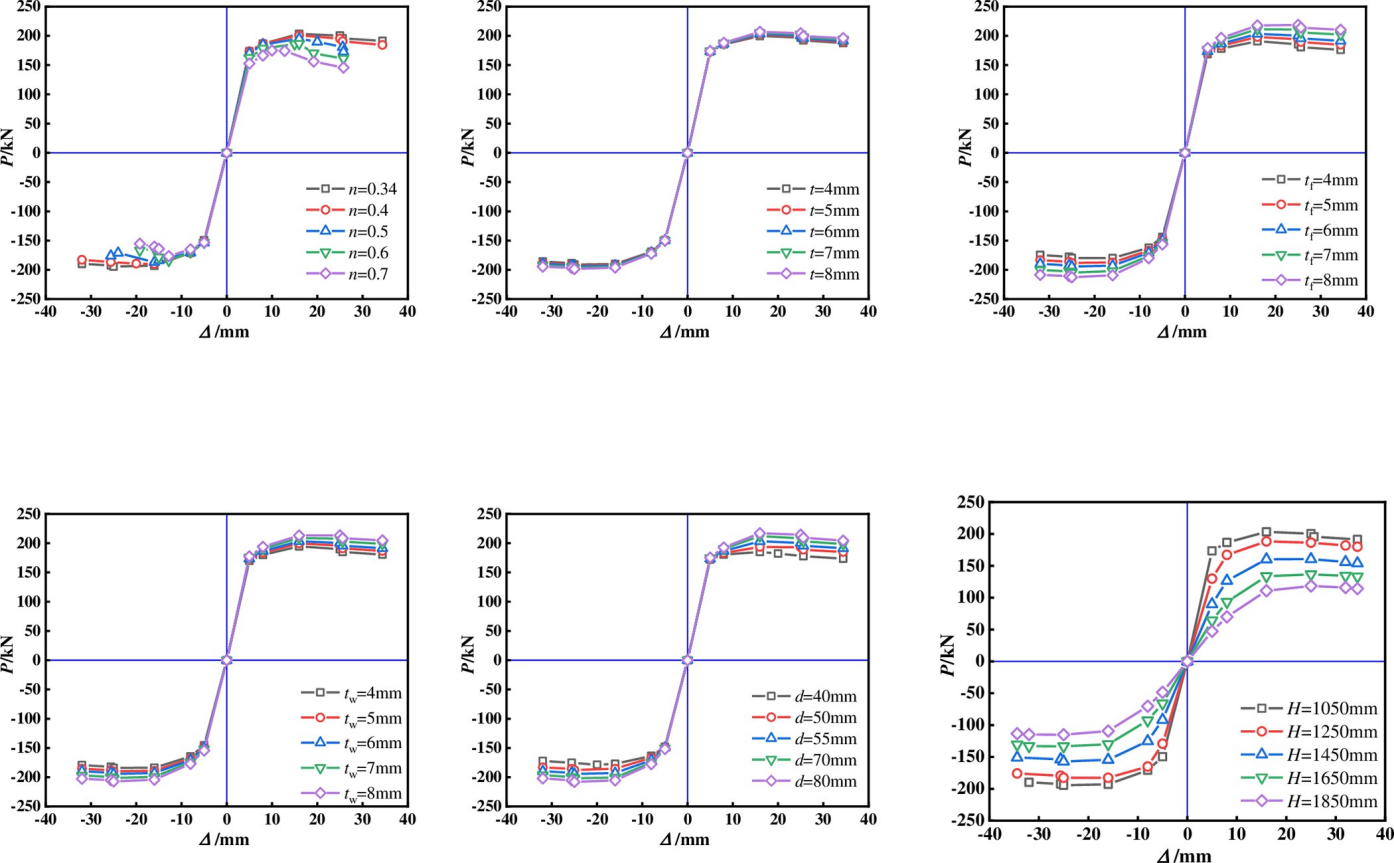
Skeleton curve. (a)Comparison of different axial compression ratios, (b)Comparison of different square steel tube thickness, (c)Comparison of different flange thickness of T-shaped steel, (d)Comparison of different web thickness of T-shaped steel, (e)Comparison of different side length of core steel tube, (f)Comparison of different height of the specimen.

Figs 9 and 10 show the hysteresis and skeleton curves of each specimen. The following conclusions can be obtained by comparing different variation parameters:

1. The ultimate bearing capacity of the specimen decreases significantly with the increase of *n*, and the envelope area of the hysteresis curve gradually decreases, with a greater rate of load decrease after reaching the peak load. This indicates that the deformation resistance of cross shaped columns is greatly affected by the *n*.
2. The shape of the hysteresis curve and skeleton curve of the specimen is less affected by changes in the thickness of the square steel tube, possibly because the square steel tube is located in the neutral axis area of the core region and participates less in the force under lateral forces.
3. With the increase of the thickness of the flange and web of T-shaped steel, the envelope area of the hysteretic curve of the specimen is larger, and the bearing capacity is also gradually increased. The shape of the skeleton curve is basically similar. It is worth noting that increasing the flange thickness has a greater impact on the hysteretic curve and skeleton curve, which indicates that increasing the flange thickness can effectively improve the seismic performance of the specimen.
4. The seismic performance of the specimen has improved with the increase of the *b*, which may be due to the increase in the core concrete area constrained by the steel tube, resulting in an increase in the strength of the concrete in the core area.
5. As the height of the specimen increases, the pinch of the hysteresis curve of the specimen becomes more severe, and the peak point and stiffness of the skeleton curve significantly decrease. The seismic performance of the specimen is significantly affected.

### Bearing capacity and ductility

**Fig 11:**
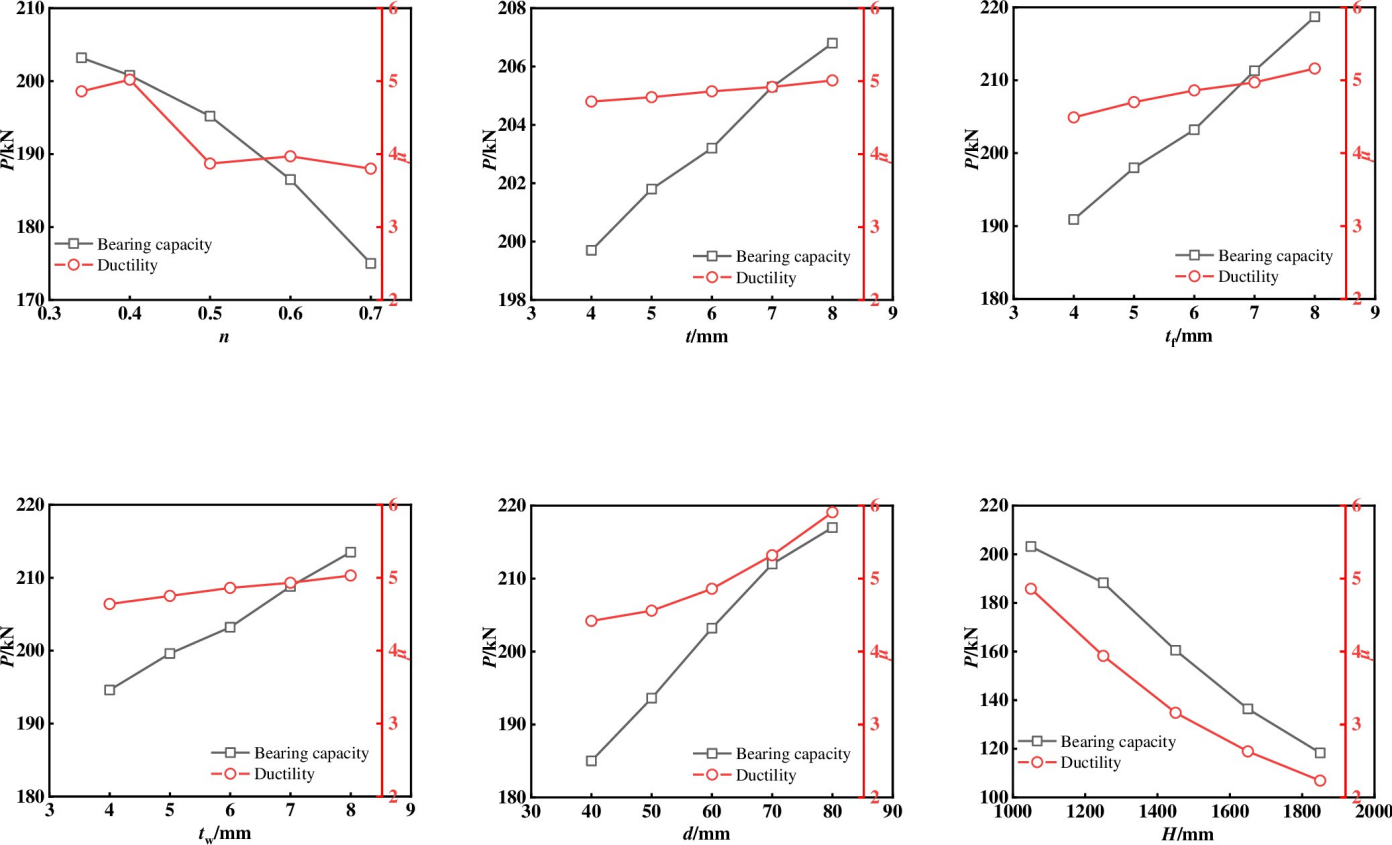
Bearing capacity and ductility. (a)Comparison of different axial compression ratios, (b)Comparison of different square steel tube thickness, (c)Comparison of different flange thickness of T-shaped steel, (d)Comparison of different web thickness of T-shaped steel, (e)Comparison of different side length of core steel tube, (f)Comparison of different height of the specimen.

The seismic mechanical performance indicators of all specimens are shown in Table 1, which are the average values under positive and negative loading. **Fig 11** shows the influence curves of different variables on bearing capacity and ductility. The following conclusions can be obtained by comparing different variation parameters:

1. The increase in *n* leads to a degradation of the ultimate bearing capacity and ductility of the specimen. It is worth noting that the overall ductility shows a trend of first increasing and then decreasing. Compared with the specimen with the *n =* 0.34, the ultimate bearing capacity of the specimen with the *n =* 0.4, 0.5, 0.6 and 0.7 decreases by 1.2%, 3.9%, 8.3% and 13.9% respectively. The ductility increases by 3.2% when the *n =* 0.4, and decreases by 20.4%, 18.4%, and 21.9% when the *n =* 0.5, 0.6, and 0.7, respectively, indicating that the axial compression ratio is the key factor affecting the seismic performance of the cross shaped concrete columns with built-in T-shaped steel and steel tubes. According to the requirements of the code for ductility coefficient greater than 3 [17], the limit value of axial compression ratio is more than 0.7, and this composite columns can still have good seismic performance under high axial compression ratio.
2. With the increase of the *t* tube, the ultimate bearing capacity of the specimen gradually increases. Compared with the specimen with the thickness of 4 mm, the ultimate bearing capacity of the specimen with the *t =* 5 mm, 6 mm, 7 mm, and 8 mm increased by 1.0%, 1.7%, 2.8%, and 3.5% respectively, while the ductility showed a trend of gradual reduction, and degraded by 1.8%, 3.0%, 4.6%, and 5.8% respectively. On the whole, the increase of the steel content in the core area is not obvious for the improvement of the bearing capacity, and reduces the ductility to a certain extent. The reason may be that the core area is in the neutral axis area, so the increase of the steel content in the area has little impact on the bearing capacity, but it increases the stiffness of the area, increases the stiffness ratio of the core area to the flange, and reduces the ductility.
3. With the increase of the *t*_f_ and *t*_w_, the ultimate bearing capacity and ductility of the specimen gradually increase. Compared with the specimen with the thickness of the *t*_f_ = 4mm, the ultimate bearing capacity of the specimen with the *t*_f_ = 5mm, 6mm, 7mm and 8mm increased by 3.7%, 6.4%, 10.6% and 14.5% respectively, and the ductility increased by 4.6%, 8.2%, 10.6% and 14.9% respectively. Compared with the specimen with the *t*_w_ = 4 mm, the ultimate bearing capacity of the specimen with the *t*_w_ = 5 mm, 6 mm, 7 mm and 8 mm increased by 2.5%, 4.4%, 7.2% and 9.7% respectively, and the ductility increased by 2.3%, 4.7%, 6.2% and 8.4% respectively. In summary, increasing the thickness of the T-shaped steel flange can better improve the seismic performance of the cross shaped column, compared to increasing the thickness of the T-shaped steel web plate. Therefore, it is suggested to improve the seismic performance of cross shaped concrete columns with built-in T-shaped steel and steel tubes by increasing the thickness of flange steel plate.
4. The ultimate bearing capacity and ductility of the cross shaped column gradually increase with the increase of the size of the square steel tube. Compared with the specimen with the *d* = 55 mm, the ultimate bearing capacity of the specimen with the *d* = 40 mm, 50 mm, 70 mm and 80 mm increased by 4.6%, 9.8%, 14.5% and 17.2% respectively, and the ductility increased by 3.1%, 9.9%, 20.3% and 33.7% respectively. The reason may be that the larger the size of the steel tube, the higher the steel content in the core area, and the larger the area of the confined concrete in the core area. According to the existing restraint theory, the strength and ductility of the concrete after being restrained have been greatly improved, so the seismic performance of this composite columns has been improved.
5. As the height of the specimen increases, the bearing capacity and ductility of the specimen significantly decrease. Compared to the specimen with a height of 1050mm, the bearing capacity and ductility decreased by 41.8% and 54.2% when the height increased to 1850mm, respectively. According to the requirement in the standard that the ductility of seismic components should be greater than 3, the critical height should be between 1450mm and 1650, and the corresponding shear span ratio should be between 4.1 and 4.7.

### Stiffness degradation

**Fig 12:**
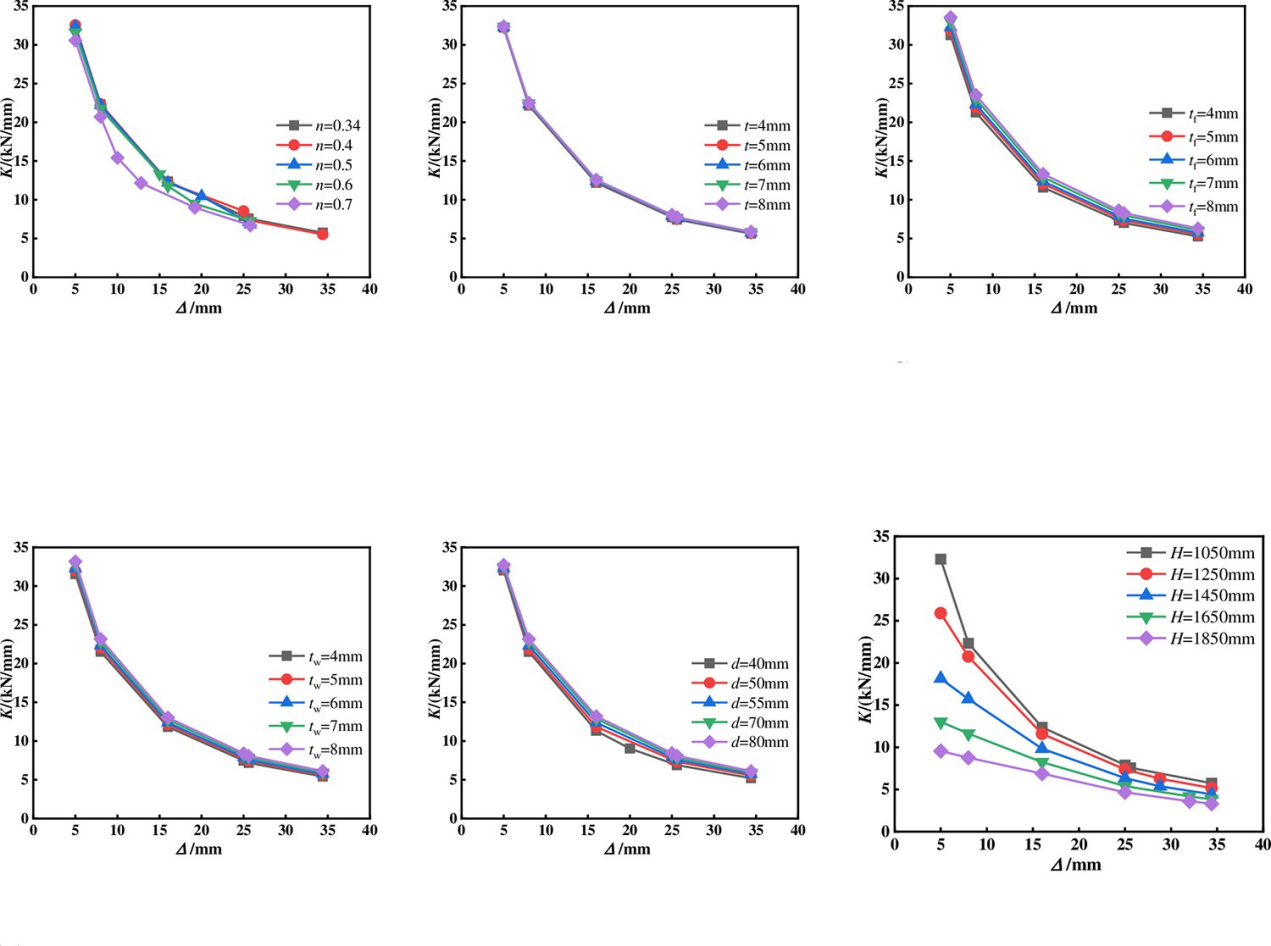
Stiffness degradation curve. (a)Comparison of different axial compression ratios, (b)Comparison of different square steel tube thickness, (c)Comparison of different flange thickness of T-shaped steel, (d)Comparison of different web thickness of T-shaped steel, (e)Comparison of different side length of core steel tube, (f)Comparison of different height of the specimen.

**Fig 12** shows the comparison of stiffness degradation curves of all specimens. The calculation method is to take the secant stiffness at the peak point of each level of the skeleton curve as the shear stiffness of that level:

$$K=\frac{\left|+P_{i}|+|-P_{i}\right|}{\left|+\Delta_{i}|+|-\Delta_{i}\right|}$$ (2)

Where, *Δ*_i_ and *P*_i_ are the maximum displacement and load corresponding to the level *i* cycle respectively;+ and—Indicates forward and reverse loading respectively.

The following conclusions can be obtained by comparing different variation parameters:

1. The shape of the stiffness degradation curve of a cross shaped column is less affected by the *n*. The greater the axial compression ratio, the greater the initial stiffness. When the horizontal displacement is greater than 8 mm, the greater the axial compression ratio, the faster the stiffness degradation rate.
2. The stiffness of the cross shaped column is not significantly affected by the *t*. It is worth noting that the stiffness degradation of the cross shaped column slows down as the size of the steel tube increases, because the increase in the size of the steel tube in the core area increases the area of the constrained concrete. According to the research results of steel tube concrete [18], its stiffness has been improved to a certain extent, therefore the shear stiffness of the specimen has been improved.
3. With the increase of the *t*_f_ and *t*_w_, the stiffness degradation of the specimen is slower, and the secant stiffness under the same horizontal displacement is greater. On the whole, increasing the thickness of the flange steel plate can more slow down the degradation of the stiffness and improve the initial stiffness.
4. As the height of the specimen increases, the initial stiffness of the specimen significantly decreases, but the shape of the stiffness degradation curve is basically similar. This is because increasing the height of the specimen weakens the bending stiffness of the component.

## Calculation method of bending capacity

According to the experimental results and FE analysis, under the combined action of constant axial pressure and low cycle repeated horizontal displacement, the cross shaped concrete column with built-in T-shaped steel and steel tubes undergoes compression-bending failure, and the failure mode and stress mechanism are similar to those of eccentric compression components. Therefore, on the basis of the theory of computation of the cross column’s eccentric compression normal section, the effect of section steel can be considered to calculate the compression and bending bearing capacity of this kind of composite column. The basic method of normal section bearing capacity of cross section column eccentrically compressed members is similar to that of rectangular section, that is, according to the concrete height of different compression areas, it is determined whether it belongs to large eccentric compression failure or small eccentric compression failure. However, due to the irregular shape of cross section, the compression concrete under large eccentric compression has two different shapes of compression areas.

**Fig 13:**
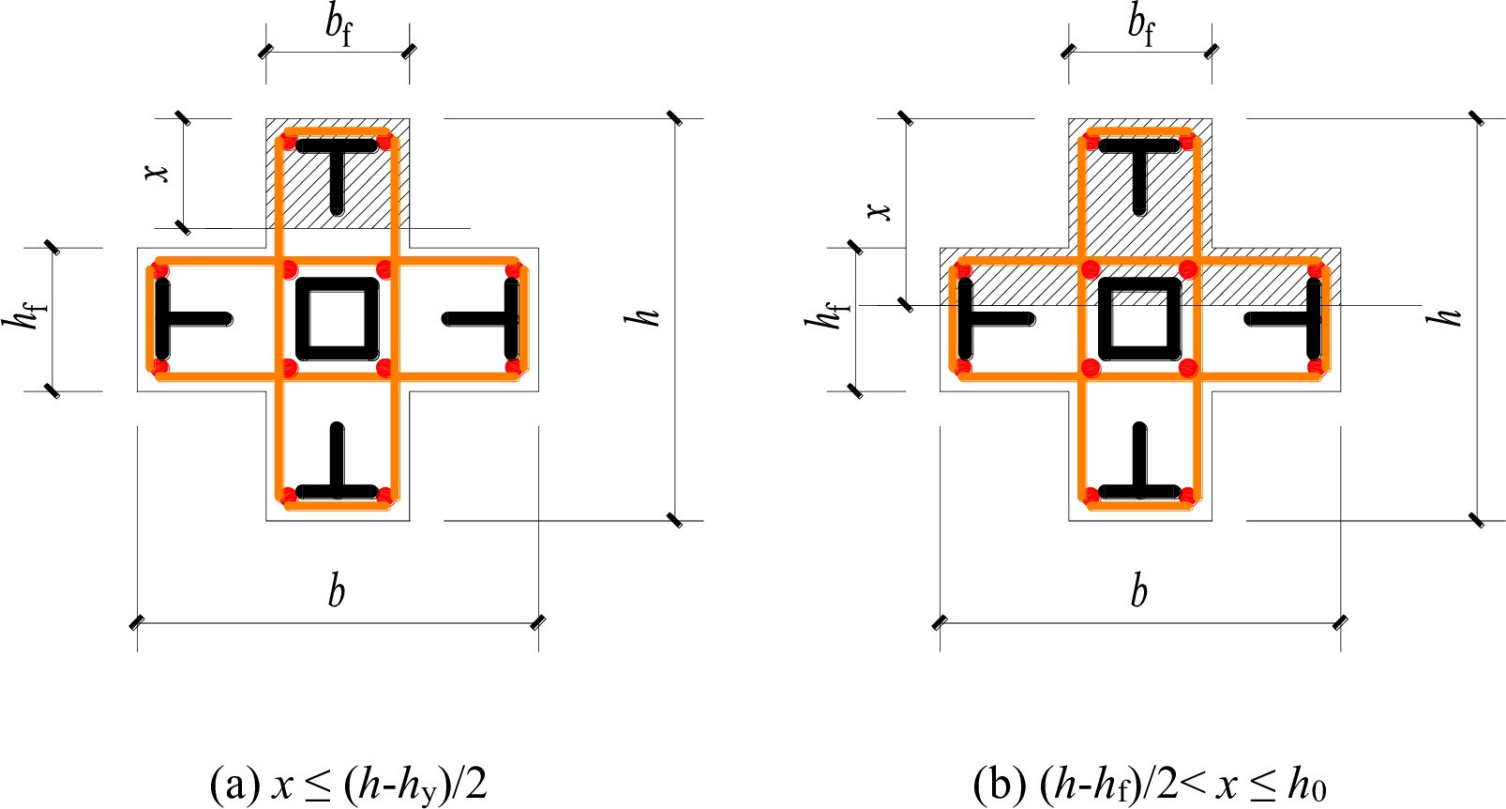
Large eccentric compression model. (a) *x* ≤ (*h*-*h*_y_)/2, (b) (*h*-*h*_f_)/2< *x* ≤ *h*_0_.

**Fig 14:**
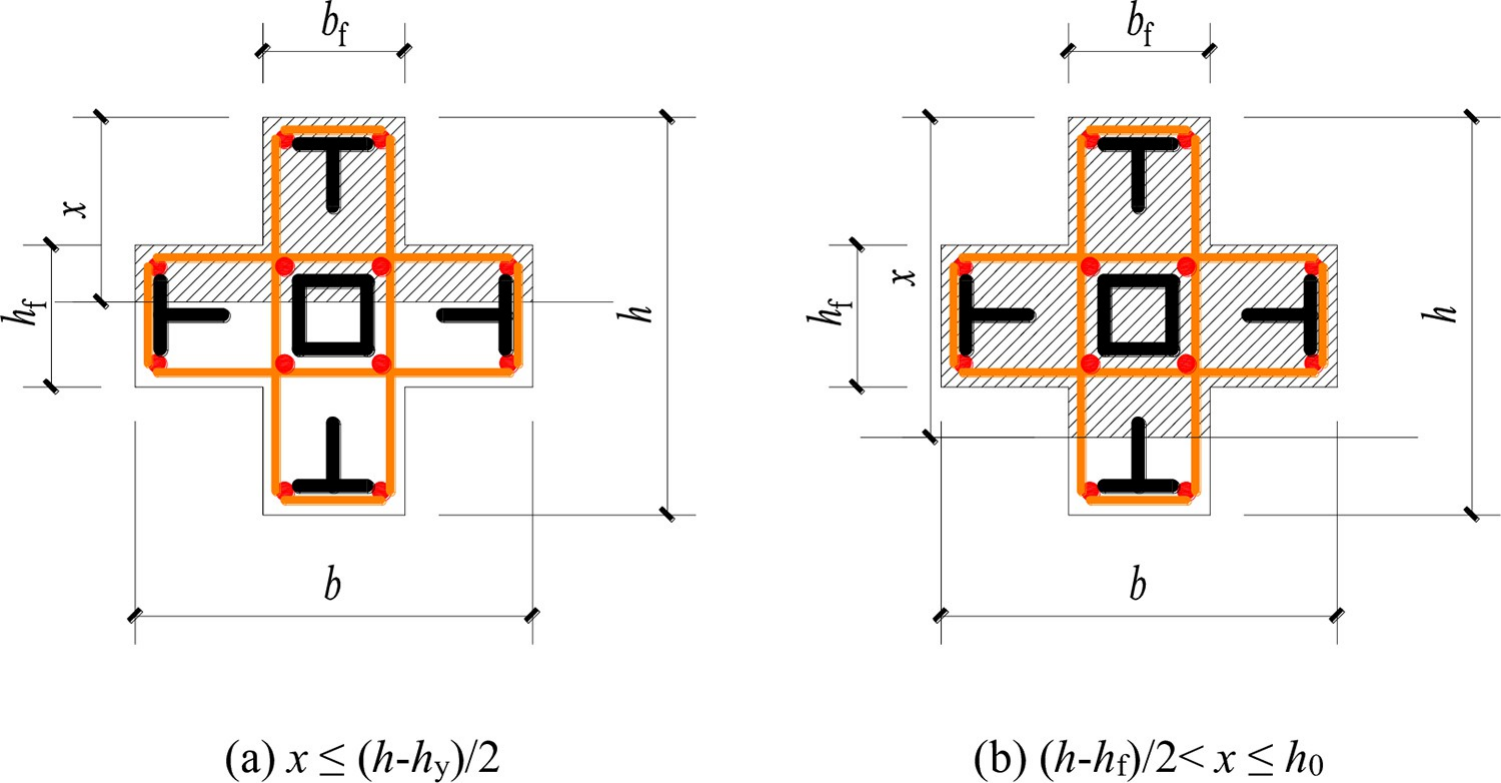
Small eccentric compression model. (a) *x* ≤ (*h*-*h*_y_)/2, (b) (*h*-*h*_f_)/2< *x* ≤ *h*_0_.

Due to the temporary blank in the standard for steel reinforced concrete special-shaped columns, ordinary reinforced concrete special-shaped columns do not consider the role of steel, so the calculation results will inevitably be too conservative. This article considers the effects of T-shaped steel and square steel tubes in the traditional calculation method of bending bearing capacity of cross shaped reinforced concrete columns, and assumes that the steel tubes in the core area do not participate in the force (because the square steel tube is located near the neutral axis), in order to make corrections. The eccentric compression model are shown in **Figs 13** and **14**.

### Large eccentric compression

(1) When the height of compression zone *x* ≤ (*h*-*h*_f_)/2, the calculation formula of rectangular section column shall be adopted:

$$N\leq \alpha_{1}f_{c}bx+f{'}_{y}A{'}_{s}+f{'}_{T}A{'}_{T}-f_{y}A_{s}-f_{T}A_{T}$$ (3)

$$Ne\leq \alpha_{1}f_{c}bx(h_{0}-\frac{x}{2})+f{'}_{y}A{'}_{s}(h_{0}-a\prime_{s})+f{'}_{T}A{'}_{T}(h_{0}-a\prime_{s})$$ (4)

(2) When the height of the compression zone (*h*-*h*_f_)/2< *x* ≤ *h*_0_, the compression zone is inverted T-shaped, and the role of the section steel is considered on the basis of reference [19]:

$$N\leq \alpha_{1}f_{c}[b_{f}x+(b‐b_{f})(x‐\frac{h‐h_{f}}{2})]+f{'}_{y}A\prime_{s}+f{'}_{T}A\prime_{T}-f_{y}A_{s}-f_{T}A_{T}$$ (5)

$$Ne\leq \alpha_{1}f_{c}[b_{f}x(h_{0}-\frac{x}{2})+(b‐b_{f})(x-\frac{h‐h_{f}}{2})(h_{0}-\frac{x}{2}-\frac{h‐h_{f}}{4})]$$ (6)

### Small eccentric compression

When the compression zone is an inverted T shape, the calculation method was consistent with Eq (3). But when the compression zone is a cross shape, the role of the section steel is considered on the basis of reference [19]:

$$N\leq \alpha_{1}f_{c}[b_{f}x+(b‐b_{f})h_{f}]+f{'}_{y}A\prime_{s}+f{'}_{T}A\prime_{T}-\sigma_{s}A_{s}-\sigma_{T}A_{T}$$ (7)

$$Ne\leq \alpha_{1}f_{c}[b_{f}x(h_{0}-\frac{x}{2})+(b‐b_{f})h_{f}(h_{0}-\frac{h}{2})]+f{'}_{y}A{'}_{s}(h_{0}-\alpha {'}_{s})+f{'}_{T}A{'}_{T}(h_{0}-\alpha {'}_{s})$$ (8)

**Fig 15:**
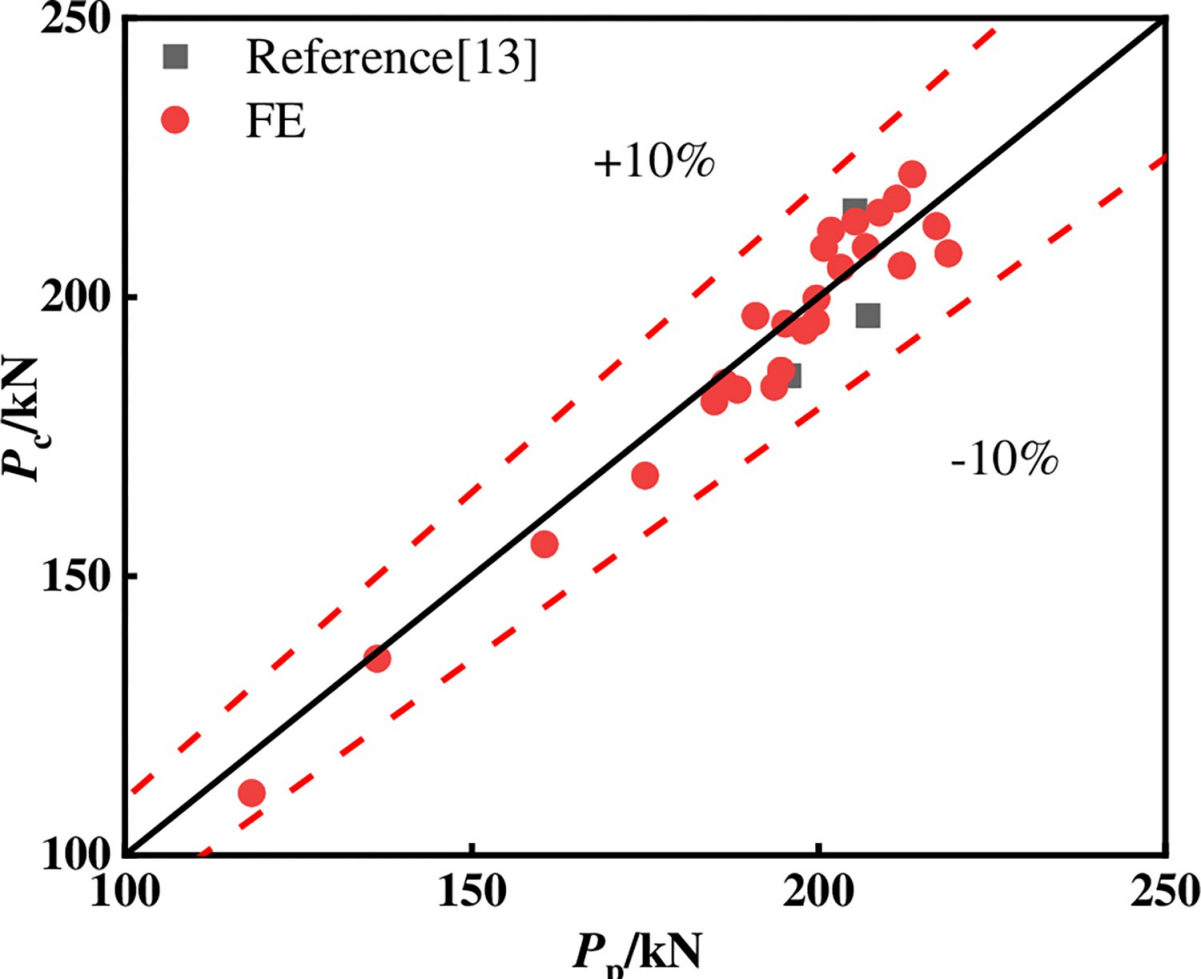
Comparison of calculation results.

Finally, the horizontal load is converted by **Eq 9**, and the FE model specimen and the specimen in reference [13] are calculated using the proposed calculation method. The comparison results are shown in **Fig 15 and Table 1**. It can be seen from the figure that the average value of the ratio between the calculation results and the test/FE results is 0.997, and the standard deviation is 0.035, which is in good agreement. The proposed calculation method is verified.


$$M=P_{\max}L+N\Delta_{\max}$$ (9)

Where, *P*_max_ is the horizontal limit load of each specimen, *L* is the net height from the base of the specimen to the action point of the column head, *N* is the axial pressure, and Δ_max_ is the horizontal displacement corresponding to the horizontal limit load.

## Conclusions

In this paper, the FE modeling verification and seismic performance parameter analysis of 25 cross shaped concrete columns with built-in T-shaped steel and steel tubes were completed, and the following conclusions were drawn:

1. ABAQUS FE analysis software was adopted to simulate and analyze the seismic performance of the cross shaped concrete columns with built-in T-shaped steel and steel tubes that have been tested. The failure mode, hysteretic curve and skeleton curve calculated by the FE method are in good agreement with the test results, and the error is within 10%.
2. The steel and steel tubes inside the cross shaped column do not yield before the specimen was in the elastic stage, and the T-shaped steel at the column base first yields after entering the plastic stage. When the cross shaped column fails, the longitudinal steel bars and T-shaped steel webs yield. The horizontal displacement continues to increase, and the square steel tube in the core area finally yields. It is recommended to increase the thickness of the T-shaped steel flange to improve the seismic performance of this type of cross shaped column.
3. The ultimate bearing capacity and ductility of the specimen decrease with the increase of axial compression ratio, but the reinforcement form of built-in T-shaped steel and square steel tubes can ensure that the ductility of the cross shaped column meets the standard requirements under high axial compression ratio. The increase in thickness of square steel tubes increases the ultimate bearing capacity of the cross shaped column, but the ductility deteriorates. The seismic performance of the cross shaped column has been improved due to the increase in the size of the steel tube, which results in an increase in the concrete area constrained by the core area. The increase in the thickness of the T-shaped steel flange has a greater improvement effect on the seismic performance of the specimen than the improvement effect of the web. An increase in the height of the specimen will significantly reduce the bearing capacity and ductility of the specimen. When the shear span ratio is not greater than 4.1, it can meet the standard requirement of ductility greater than 3 for seismic components.
4. Based on the existing specifications and documents, a modified method for calculating the bending capacity of the cross shaped concrete columns with built-in T-shaped steel and steel tubes is proposed, with an error of less than 5%.
